# Subtyping of *Cryptosporidium cuniculus* and genotyping of *Enterocytozoon bieneusi* in rabbits in two farms in Heilongjiang Province, China

**DOI:** 10.1051/parasite/2016063

**Published:** 2016-11-24

**Authors:** Ziyin Yang, Wei Zhao, Yujuan Shen, Weizhe Zhang, Ying Shi, Guangxu Ren, Di Yang, Hong Ling, Fengkun Yang, Aiqin Liu, Jianping Cao

**Affiliations:** 1 Department of Parasitology, Harbin Medical University Harbin Heilongjiang 150081 China; 2 National Institute of Parasitic Diseases, Chinese Center for Disease Control and Prevention, Key Laboratory of Parasite and Vector Biology, Ministry of Health, WHO Collaborating Centre for Malaria, Schistosomiasis and Filariasis Shanghai 200025 China

**Keywords:** rabbits, *Cryptosporidium*, *Enterocytozoon bieneusi*, ITS region, SSU rDNA, *gp*60 gene

## Abstract

*Cryptosporidium* spp. and *Enterocytozoon bieneusi* are two prevalent opportunistic pathogens in humans and animals. Currently, few data are available on genetic characterization of both pathogens in rabbits in China. The aim of the present study was to understand prevalence and genetic characterization of *Cryptosporidium* spp. and *E. bieneusi* in rabbits. We collected 215 fecal samples from 150 Rex rabbits and 65 New Zealand White rabbits on two different farms in Heilongjiang Province, China. *Cryptosporidium* spp. and *E. bieneusi* were tested by polymerase chain reaction (PCR) and sequencing the partial small subunit of ribosomal DNA (SSU rDNA) and the internal transcribed spacer (ITS) region of rDNA, respectively. *Cryptosporidium* was detected in 3.3% (5/150) of Rex rabbits and 29.2% (19/65) of New Zealand White rabbits. All the 24 *Cryptosporidium* isolates were identified as *C. cuniculus*. *Enterocytozoon bieneusi* was only found in 14.7% (22/150) of Rex rabbits. Five known genotypes: CHN-RD1 (*n* = 12), D (*n* = 3), Type IV (*n* = 2), Peru6 (*n* = 1), and I (*n* = 1), and three novel ones CHN-RR1 to CHN-RR3 (one each) were detected. By analyzing the 60-kDa glycoprotein (*gp*60) gene sequences of *C. cuniculus* isolates, three subtypes were obtained: VbA28 (*n* = 2), VbA29 (*n* = 16), and VbA32 (*n* = 3). All these three *C. cuniculus* subtypes were reported previously in humans. Four known *E. bieneusi* genotypes have been found to be present in humans. The three novel ones fell into zoonotic group 1. The results suggest zoonotic potential of *C. cuniculus* and *E. bieneusi* isolates in rabbits.

## Abstract (fr)

*Cryptosporidium* spp. et *Enterocytozoon bieneusi* sont deux agents pathogènes opportunistes répandus chez les humains et les animaux. Actuellement, peu de données sont disponibles sur la caractérisation génétique de ces deux agents pathogènes chez les lapins en Chine. Le but de la présente étude était de comprendre la prévalence et la caractérisation génétique des *Cryptosporidium* spp. et *E. bieneusi* chez les lapins. Nous avons recueilli 215 échantillons fécaux de 150 lapins Rex et 65 lapins blancs de Nouvelle Zélande dans deux fermes différentes dans la province du Heilongjiang, en Chine. *Cryptosporidium* spp. et *E. bieneusi* ont été testés par PCR et respectivement par séquençage partiel de la petite sous-unité de l’ADN ribosomal (SSU-ADNr) et de la région ITS de l’ADNr. *Cryptosporidium* a été détecté dans 3.3 % (5/150) des lapins Rex et 29.2 % (19/65) des lapins blancs de Nouvelle-Zélande. Tous les 24 isolats de *Cryptosporidium* ont été identifiés comme *C. cuniculus*. *Enterocytozoon bieneusi* n’a été trouvé que dans 14.7 % (22/150) des lapins Rex. Cinq génotypes connus : CHN-RD1 (*n* = 12), D (*n* = 3), Type IV (*n* = 2), Peru6 (*n* = 1) et I (*n* = 1), et trois nouveaux, CHN-RR1 à CHN-RR3 (un de chaque) ont été détectés. En analysant les séquences du gène de la glycoprotéine de 60 kDa (*gp*60) des isolats de *C. cuniculus*, trois sous-types ont été obtenus: VbA28 (*n* = 2), VbA29 (*n* = 16) et VBA32 (*n* = 3). Ces trois sous-types de *C. cuniculus* ont été rapportés auparavant chez l’homme. Quatre génotypes d’E*. bieneusi* connus ont été rapportés chez les humains. Les trois nouveaux appartiennent au groupe zoonotique 1. Les résultats suggèrent un potentiel zoonotique des isolats de *C. cuniculus* et *E. bieneusi* des lapins.

## Introduction

*Cryptosporidium* spp. and *Enterocytozoon bieneusi* are obligate intracellular eukaryotes, and both of them can infect the intestine of hosts*.* Clinical symptoms of human disease caused by either *Cryptosporidium* or *E. bieneusi* are variable, ranging from asymptomatic infection or self-limiting diarrhea in healthy people to chronic or life-threatening diarrhea in immunocompromsied individuals [33, 48]. Besides humans, these microorganisms have also been found in numerous animal species [33, 41].

*Cryptosporidium* is a complex genus. To date, 30 *Cryptosporidium* species and more than 40 genotypes have been described, and among them, 20 *Cryptosporidium* species/genotypes have been reported in humans, with *C. hominis* and *C. parvum* responsible for the majority of infections [15, 23, 28, 41, 42]. For *E. bieneusi*, more than 240 ITS genotypes have been identified worldwide [18, 45], and at least 70 genotypes have been found in humans, with 33 genotypes being zoonotic [21, 33, 61]. In phylogenetic analysis, all the published genotypes belong to nine distinct groups. Group 1 is composed of the common zoonotic genotypes and groups 2–9 mostly contain host-adapted genotypes [14, 19, 33]. The findings of the same species/genotypes of the two pathogens in humans and animals support presumption of zoonotic potential [33, 41].

Currently, fumagillin is effective in the treatment of microsporidiosis caused by *E. bieneusi* [2], while nitazoxanide has an effect on cryptosporidiosis in non-HIV patients to a certain extent [41]. However, understanding *Cryptosporidium* and *E. bieneusi* epidemiology in different host species is still a key step to prevent *Cryptosporidium* and *E. bieneusi* infections in humans, particularly in determining the zoonotic potential of animal-derived isolates.

In China, Rex rabbits are one of the most common farmed animal species used for fur and meat production; and with the development of the fur industry in recent years, the number of Rex rabbits has been increasing. New Zealand White rabbits are mainly used for food production and experiments. The aim of the present study was to understand the prevalence of natural infection and genetic characterization of *Cryptosporidium* and *E. bieneusi* in Rex rabbits and New Zealand White rabbits. In addition, the zoonotic potential of *Cryptosporidium* and *E. bieneusi* isolates was assessed.

## Materials

### Ethics statement

Before beginning this study, we described the protocol to the farm managers and obtained their permission. In this study, only fecal samples of the farm animals were collected. Meanwhile, the study protocol was reviewed and approved by the Research Ethics Committee and the Animal Ethics Committee of Harbin Medical University. The work concerning animals strictly followed guidelines in accordance with the Regulations for the Administration of Affairs Concerning Experimental Animals.

### Specimen collection

During the period from March 2015 to February 2016, a total of 215 rabbit fecal samples were collected from Heilongjiang Province, China, including 150 from Rex rabbits on a farm in Huaqiang Fur Breeding Base in Bayan County and 65 from New Zealand White rabbits on a farm in Huaxing Breeding Base of Rabbits in Harbin City. One fresh fecal specimen (approximately 15 g) of each animal was collected. All the fecal specimens were transported to our laboratory in a cooler with ice packs within 24 h and stored in refrigerators at 4 °C until molecular analysis. Rex rabbits and New Zealand White rabbits were collected from the two different farms, both accounting for approximately 5% of the total animals. Rex rabbits and New Zealand White rabbits were five or six and two or three months old, respectively. No apparent clinical signs of diarrhea were observed at the time of sampling.

### DNA extraction

To reduce interference from crude fiber and impurities in rabbit manure, the fecal specimens were sieved and washed with distilled water by centrifugation for 10 min at 1500 g×. Genomic DNA was extracted from 180–200 mg washed fecal pellets using a QIAamp DNA Stool Mini Kit (QIAgen, Hilden, Germany) according to manufacturer-recommended procedures. DNA was eluted in 200 μL of AE buffer and stored at −20 °C in a freezer prior to polymerase chain reaction (PCR) analysis.

### *Cryptosporidium* genotyping and subtyping

All genomic DNA samples were subjected to nested PCR targeting *Cryptosporidium* by amplification of an 830 bp nucleotide fragment of the small subunit (SSU) rDNA of *Cryptosporidium*. The primers and the cycling parameters in PCR analysis were used as previously described by Xiao et al. [52]. Subtyping of *Cryptosporidium*-positive samples was performed by nested PCR amplification of an approximately 800–850 bp fragment of the *gp*60 gene [1].

### *E. bieneusi* genotyping

To identify the presence and genotypes of *E. bieneusi*, all the genomic DNA samples were subjected to nested PCR amplification of a 389 bp nucleotide fragment of the rDNA of *E. bieneusi* containing 76 bp of the 3′ end of SSU rDNA, 243 bp of the internal transcribed spacer (ITS) region, and 70 bp of the 5′ region of the large subunit (LSU) rDNA [7]. All the genotypes were named based on 243 bp of the ITS region of *E. bieneusi* according to the established nomenclature system [44].

### Nucleotide sequencing and analyzing

All the secondary PCR products of the expected size were directly sequenced with primers used for the secondary PCR after being purified on an ABI PRISM 3730XL DNA Analyzer by Sinogeno- max Biotechnology Co. Ltd. (Beijing, China), using the BigDye Terminator v3.1 Cycle Sequencing Kit (Applied Biosystems, USA). Sequence accuracy was confirmed by two-directional sequencing and by sequencing a new PCR product if necessary for some DNA samples, from which novel nucleotide sequences were obtained. Nucleotide sequences obtained in the present study were subjected to BLAST searches (http://www.ncbi.nlm.nih.gov/blast/), and then aligned and analyzed with each other and reference sequences from GenBank using Clustal X 1.81 (http://www.clustal.org/).

### Phylogenetic analysis

Phylogenetic relationships among subtypes of *Cryptosporidium* and genotypes of *E. bieneusi* determined in the present study with those presently available in public databases were explored using the Mega 5 program (http://www.megasoftware.net/) to construct two neighbor-joining trees based on the evolutionary distances calculated by the Kimura 2-parameter model. The reliability of the trees was assessed using the bootstrap analysis with 1000 replicates.

## Results

### Infection rates of *Cryptosporidium* and *E. bieneusi*

*Cryptosporidium* was detected in the two rabbit breeds by nested PCR amplification of the SSU rDNA. Infection rates of *Cryptosporidium* were 3.3% (5/150) and 29.2% (19/65) in Rex rabbits and New Zealand White rabbits, respectively. There was a significant difference in infection rates of *Cryptosporidium* between the two rabbit breeds by means of a χ^2^ test (χ^2^ = 30.67, *p* < 0.01) (Table 1).

**Table 1.** Prevalences and genotypes/subtypes of *C. cuniculus* and *E. bieneusi* in rabbits.

Host	Sample (*n*)	*C. cuniculus*		*E. bieneusi*	
		No. of positive (%)	*Gp*60 subtype (*n*)	No. of positive (%)	ITS genotypes (*n*)
Rex rabbits	150	5 (3.3)	VbA32 (3)	22 (14.7)	CHN-RD1 (12); D (3); Type IV (2); Peru6 (1); I (1); CHN-RR1 to CHN-RR3 (1 each)
New Zealand White rabbits	65	19 (29.2)	VbA29 (16); VbA28 (2)	–	–

Note: The bars denote negative results.

By amplifying the ITS region of the rDNA, *E. bieneusi* was detected in 14.7% (22/150) of Rex rabbits, and there was an absence of *E. bieneusi* in New Zealand White rabbits.

### *Cryptosporidium* species and subtypes

**Figure 1.**
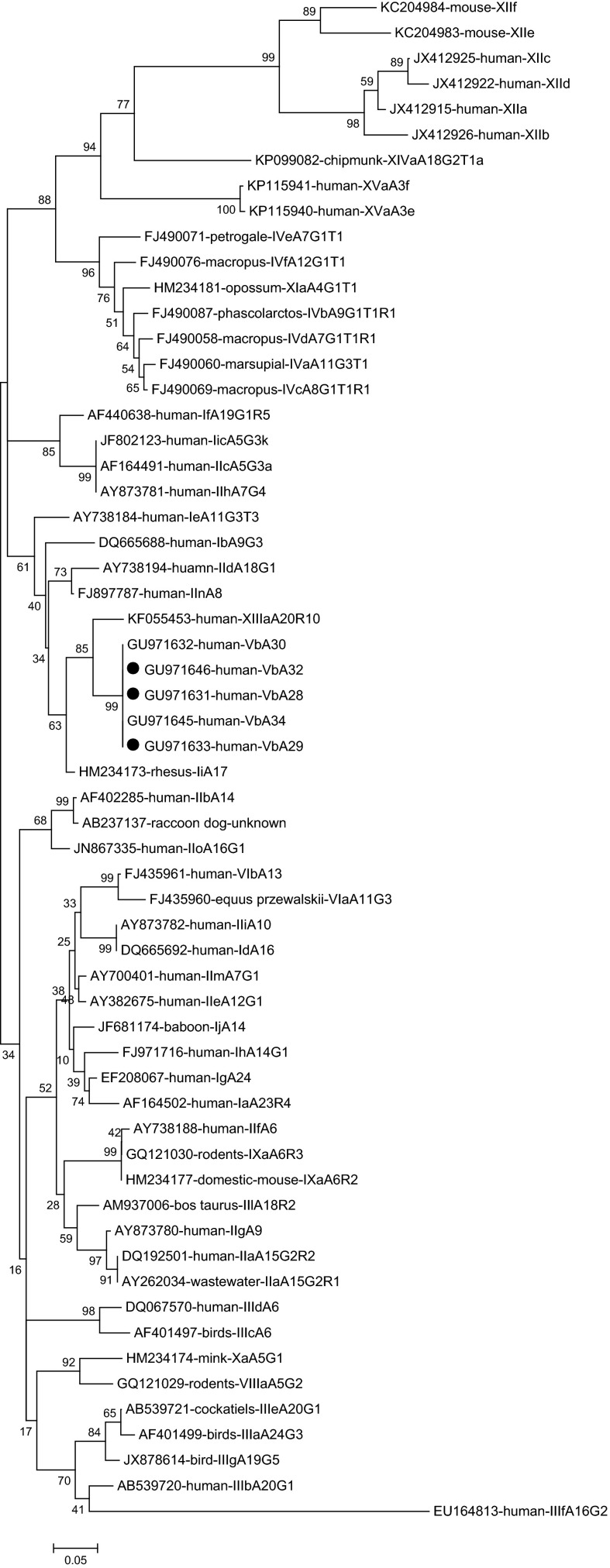
Phylogenetic relationship of *gp*60 subtypes of *Cryptosporidium* species/genotypes. The relationships between *C. cuniculus* subtypes identified in the present study and known subtypes of other *Cryptosporidium* species/genotypes deposited in the GenBank were inferred by a neighbor-joining analysis of *gp*60 gene sequences based on genetic distance by the Kimura 2-parameter model. The numbers on the branches are percent bootstrapping values from 1000 replicates. Each sequence is identified by its accession number, host origin, and subtype designation. The circles filled in black indicate the subtypes identified in this study.

Analysis of 24 sequences of the SSU rDNA of *Cryptosporidium* showed that all the *Cryptosporidium* isolates were identical to each other and had 100% similarity with a *C. cuniculus* isolate from a rabbit (HQ397716). *C. cuniculus*-positive specimens were further subtyped by amplifying the *gp*60 gene. Only 21 *C. cuniculus* isolates produced the expected PCR product and were successfully sequenced. Three subtypes were observed according to established nomenclature [39]: VbA28 (*n* = 2), VbA29 (*n* = 16) and VbA32 (*n* = 3). Two subtypes VbA28 and VbA29 were identified in New Zealand White rabbits while one subtype VbA32 in Rex rabbits (Table 1). Phylogenetic analysis of the *gp*60 nucleotide sequences revealed that C. *cuniculus* subtypes VbA28, VbA29 and VbA32 fell into the same clade as *C. erinacei* subtype XIIIaA20R10, and were genetically close to *C. parvum* subtype families IId and IIn, and *C. hominis* subtype families Ib and Ii (Fig. 1).

### *E. bieneusi* genotypes

By nucleotide sequence analysis of the ITS region of the rDNA of *E. bieneusi*, eight genotypes were identified in the Rex rabbits, including five known genotypes (CHN-RD1, Type IV, Peru6, D, and I) and three novel ones (CHN-RR1, CHN-RR2, and CHN-RR3) (Table 1). Genotype CHN-RD1 was found in 54.5% (12/22) of *E. bieneusi* isolates, showing dominance. The remaining genotypes were all at a low frequency: 13.6% (3/22) for genotype D, 9.1% (2/22) for genotype Type IV, and 4.5% (1/22) for genotypes I, CHN-RR1, CHN-RR2, and CHN-RR3.

**Figure 2.**
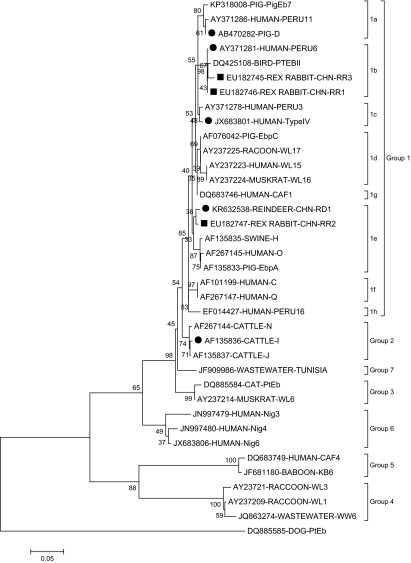
Phylogenetic relationships of *Enterocytozoon bieneusi* genotype groups. The relationships between *Enterocytozoon bieneusi* genotypes identified in the present study and other known genotypes deposited in the GenBank were inferred by a neighbor-joining analysis of ITS sequences based on genetic distance by the Kimura 2-parameter model. The numbers on the branches are percent bootstrapping values from 1000 replicates. Each sequence is identified by its accession number, host origin, and genotype designation. The group terminology for the clusters is based on the work of Zhao et al. [60]. The squares and circles filled in black indicate novel and known genotypes identified in this study, respectively.

Genotype CHN-RR1 (KU182745) had two base deletions at nucleotide sites 51 and 52 of the ITS region. Genotypes CHN-RR2 (KU182746) and CHN-RR3 (KU182747) were observed to have one and two single-nucleotide polymorphisms compared to genotype G (AF135834) and genotype Peru6 (JF927955), respectively. In phylogenetic analysis, all the genotypes except genotype I (clustered into group 2) here belonged to zoonotic group 1: genotypes Peru6, CHN-RR1, and CHN-RR3 in subgroup 1b; genotype D in subgroup 1a; genotype Type IV in subgroup 1c; and genotypes CHN-RD1 and CHN-RR2 in subgroup 1e (Fig. 2).

## Discussion

In the present study, *Cryptosporidium* was identified in the two rabbit breeds, with overall prevalence of 11.2% (24/215). Epidemiological data indicate that *Cryptosporidium* has previously been identified in rabbits in Japan, with 30.3% (20/66) in dead juvenile animals and 3.33% (1/30) in healthy animals [47], and in Australia (6.8%; 12/176) [35] as well as in some areas of China:Sichuan (1.03%; 3/290), Heilongjiang (2.38%, 9/378), and Henan (3.4%, 37/1081) [30, 46, 55]. In fact, *Cryptosporidium* in farm rabbits is not often recognized due to a low prevalence and asymptomatic course of infection. However, there is an outbreak of massive mortality among farm rabbits associated with *Cryptosporidium* infection in Poland [20]. Here, *E. bieneusi* was only found in 14.7% (22/150) of Rex rabbits, and the prevalence was higher than that (0.94%; 4/426) reported in a recent study in China [57]. To our best knowledge, there are another two studies reporting *E. bieneusi* infections in rabbits, where only a small number of rabbits were involved [11, 13]. The present finding of C*ryptosporidium* and *E. bieneusi* in the asymptomatic rabbits emphasized the importance of epidemiological investigations of the two pathogens in these animals.

By sequence analysis of the partial SSU rDNA, all 24 PCR specimens positive for *Cryptosporidium* were identified as *C. cuniculus*. *C. cuniculus* is one of the zoonotic *Cryptosporidium* species, which has been strongly linked to human cryptosporidiosis [41]. Molecular epidemiological data of human cryptosporidiosis have presented increasing occurrence of *C. cuniculus* in humans in some regions/countries. It was even considered to be the third most common *Cryptosporidium* species in clinical patients with cryptosporidiosis during the 2007–2008 period in the UK [10], including a waterborne outbreak of human cryptosporidiosis caused by *C. cuniculus* from a wild rabbit entering a treated tank [9]. In fact, some sporadic human cases have been reported in Nigeria, Australia, France, and Spain [3, 22, 32, 34]. Currently, natural infection of *C. cuniculus* has only been reported in rabbits and humans, as well as a kangaroo [22]. Rabbits have been confirmed to be the main hosts of natural infection of *C. cuniculus.* To date, experimental infections have only been established in weanling rabbits (*Oryctolagus cuniculus*), immunosuppressed Mongolian gerbils (*Meriones unguiculatus*), and immunosuppressed adult Porton strain mice (*Mus musculus*) [40]. *C. cuniculus* seems to have a narrow host range. The true host range of *C. cuniculus* needs to be confirmed by subsequent molecular epidemiological studies of *Cryptosporidium*. *Gp*60 gene sequencing is the most commonly used tool for *Cryptosporidium* subtyping, aimed at identifying infection sources, investigating transmission dynamics, and understanding genetic diversity within and between *Cryptosporidium* species/genotypes as well as their taxonomy [41]. In the present study, *gp*60-based subtyping of *C. cuniculus* isolates was achieved and three subtypes were identified: VbA28, VbA29, and VbA32. The observation that all the three subtypes here have been detected in humans [10] suggests their significance in public health. Here, subtype VbA28 was found in rabbits for the first time. Actually, two different families (Va and Vb) have been described within *C. cuniculus*. To date, nine and 20 subtypes have been found in the two different subtype families, respectively [5, 9, 10, 20, 22, 30, 35, 36, 40, 46, 55] (Table 2). Based on the data summarized in Table 2, the subtypes in the Va family are mostly found in humans and occasionally seen in rabbits; in contrast, the subtypes in the Vb family appear to be more common in rabbits than in humans. By phylogenetic analysis, the subtypes in the Vb family fell into a clade with *C. parvum* and *C. hominis* (Fig. 1). In a previous phylogenetic analysis of the SSU rDNA and *hsp*70 genes, *C. cuniculus* was observed to be genetically most closely related to *C. hominis*, sharing 99.2% similarity with *C. hominis* at the SSU rDNA locus and 99.7% similarity with *C. hominis* at the *hsp*70 locus [43]. In another study, on the basis of a distance matrix derived from the alignment utilized herein to construct the phylogeny, *C. cuniculus* was reported to be genetically closest to *C. parvum* and *C. hominis*, sharing 98% similarity with each of them [35].

**Table 2.** Subtypes of *C. cuniculus* in humans and rabbits worldwide.

Host	Country	Va family	Vb family	Ref.
Human	Australia		VbA25	[22]
	UK	VaA9; VaA11; VaA18; VaA19; VaA21; VaA22; VaA23	VbA20; VbA22; VbA25; VbA26; VbA28; VbA29; VbA30; VbA32; VbA33; VbA34; VbA36; VbA37	[10, 40]
		VaA18; VaA22		[9]
		VaA18; VaA22; VaA23; VaA32		[5]
Rabbit	Australia		VbA23R3; VbA26R4	[35]
			VbA22R4; VbA23R3; VbA24R3; VbA25R4; VbA26R4	[36]
	China	VaA31		[30]
			VbA21; VbA32	[55]
			VbA36; VbA35; VbA29	[46]
			VbA28, VbA29 and VbA32	This study
	Czech Republic	VaA19		[9]
	Poland		VbA24	[20]
	UK	VaA18		[9]

By sequence analysis of the ITS region of the rDNA, eight genotypes were identified out of 22 *E. bieneusi* isolates, including five known and three novel isolates (Table 1). Genotype CHN-RD1 showed predominance in Rex rabbits (54.5%; 12/22). This genotype was originally detected in reindeers living in the northeast forest region of Great Hinggan Mountains, China [29]. The other four known genotypes (Type IV, Peru6, D, and I) have previously been found in both humans and animals, suggesting possible zoonotic transmission from Rex rabbits to humans [33, 45]. Genotypes D and Type IV are currently the two most common genotypes diagnosed in human cases of microsporidiosis caused by *E. bieneusi* [33]. Genotype D has the widest geographical distribution and animal host range [45, 60]. In China, both genotypes have been identified in children, and in HIV-positive and HIV-negative patients [50, 51, 53]. Meanwhile, they have also been found in nonhuman primates, pigs, deer, foxes, raccoon dogs, dogs, cats, rabbits, squirrels, chinchillas, snakes, Siberian tigers, lions, hippopotamus, common cranes, a red-crowned crane, a Fischer’s lovebird, and some other captive wildlife, as well as in wastewater and lake water [12, 16–18, 24–27, 38, 54, 58, 59, 61, 63]. Compared to genotypes D and Type IV, genotypes Peru6 and I have a small number and a limited geographical area in human cases infected with *E. bieneusi*, with the former only found in Peru and Portugal [4, 8, 31, 49], and the latter only in China [56]. In China, to date, genotype Peru6 has been identified in sheep and goats, reindeers, red-crowned cranes, ducks, geese, and pigeons, as well as in wastewater [25, 29, 62, 63]; genotype I has been found in nonhuman primates, cats, a chicken, pigeons, pigs, and golden takins [13, 16, 17, 37, 39, 59, 60].

In a phylogenetic analysis, all the three novel genotypes fell into zoonotic group 1. Group 1 is reported to contain 94% of the published ITS sequences of *E. bieneusi* and almost all the human-pathogenic genotypes are in this group [33]. Thus, the novel genotypes obtained here may have a large zoonotic potential.

In the present study, it was observed that there were only 241 bp in the ITS region of novel genotype CHN-RR1. In fact, length variation of the ITS region of the rDNA of *E. bieneusi* has been found: 241 bp for genotypes CHN3, CHN4 and CHN5 from children in China [56], and 242 bp for genotype CAF4 from a human in Gabon [6]. The ITS region is 243 bp in length for the vast majority of *E. bieneusi* genotypes.

### Conclusion

Our present study demonstrated an occurrence of *C. cuniculus* and *E. bieneusi* in rabbits in Heilongjiang Province, China. All the *C. cuniculus* subtypes have previously been reported in humans. All the known *E. bieneusi* genotypes except CHN-RD1 are human-pathogenic, with all the novel ones falling into zoonotic group 1. The facts above suggest zoonotic potential of *C. cuniculus* and *E. bieneusi* isolates in these animals. It is therefore necessary to make farmers and veterinarians aware of the potential for zoonotic transmission of cryptosporidiosis and microsporidiosis as a result of close contact with infected rabbits.

## Conflict of interest

The authors declare that they have no conflict of interest in relation with this paper.
